# Modeling predicts that parameters shaping action potentials and synaptic responses differ in pyramidal neurons of the visual and prefrontal cortices

**DOI:** 10.1186/1471-2202-13-S1-P93

**Published:** 2012-07-16

**Authors:** Christina M Weaver, Aniruddha Yadav, Joseph M Amatrudo, Patrick R Hof, Jennifer I Luebke

**Affiliations:** 1 Department of Mathematics, Franklin and Marshall College, Lancaster, PA 17604, USA; 2 Department of Neuroscience and Friedman Brain Institute, Mount Sinai School of Medicine, New York, NY 10029, USA; 3 Department of Anatomy and Neurobiology, Boston University School of Medicine, Boston, MA 02118, USA

## 

Pyramidal neurons in the prefrontal cortex integrate inputs that are greater in number and diversity than pyramidal neurons of the primary visual cortex [1]. We have recently characterized the morphology and physiology of layer 3 neurons from area 46 of the dorsolateral prefrontal cortex (dlPFC) and from visual area V1 in the rhesus monkey. Ultra-high resolution confocal imaging and 3D reconstruction revealed that the dendritic arbors of V1 neurons are much smaller and less complex than those of the dlPFC, and possess far fewer dendritic spines. Physiologically, whole cell patch clamp recordings demonstrated that V1 neurons have twice as high mean input resistance and significantly increased action potential firing rates compared to dlPFC neurons. Further, AMPAR-mediated spontaneous excitatory postsynaptic currents (sEPSCs) exhibit significantly faster kinetics and smaller mean amplitudes in V1 compared to dlPFC neurons. We have used compartment modeling with the NEURON simulation environment [2] to investigate the extent to which morphology accounts for the observed physiological differences. We first applied a relatively simple model, including only Hodgkin-Huxley sodium and potassium currents [3], to representative 3D reconstructions of neurons from dlPFC and V1. This model predicts that morphology alone leads to major differences in the attenuation of electrical signals coming into, and leaving, the soma in neurons from the two brain areas. It also predicts that morphology largely accounts for the increased excitability of V1 cells, but not the differences in kinetics and amplitudes of sEPSCs. The model predicts that parameters controlling passive voltage spread, the density of Hodgkin-Huxley channels, and synaptic inputs likely differ between dlPFC and V1 neurons. Yet, the simple model on which these predictions are based reproduces neither the H-current dependent ‘sag’ response during hyperpolarization, nor spike frequency adaptation, both of which are observed in dlPFC and V1 neurons. We have extended the model to include the H current [4], plus a calcium-dependent potassium channel with an associated one-pool source of intracellular calcium [5]. We have tuned the model with a combination of manual and automatic optimization techniques to match the resting potential, input resistance, and overall firing rates of the recorded dlPFC and V1 neurons. As in the simpler model, we find that morphology alone cannot account for observed physiological differences. Therefore, we predict that parameters shaping action potentials and synaptic input differ between dlPFC and V1.
